# Evaluation of problem-based learning for pharmacology based on a comprehensive analysis in undergraduate students

**DOI:** 10.1097/MD.0000000000039376

**Published:** 2024-10-11

**Authors:** Xiding Yang, Zehua Yang, Sijia Ma, Miao Yan, Yongyu Yang

**Affiliations:** a Department of Pharmacy, The Second Xiangya Hospital of Central South University, Changsha, China; b Hunan Drug Inspection Center, Changsha, China; c Hunan Provincial Engineering Research Central of Translational Medical and Innovative Drug, The Second Xiangya Hospital of Central South University, Changsha, China.

**Keywords:** meta-analysis, pharmacology, problem-based learning, undergraduate

## Abstract

**Background:**

Problem-based learning (PBL) has been widely employed in pharmacology teaching. However, the benefits of PBL for undergraduate students have not been clearly demonstrated. We performed a meta-analysis to compare the effects of PBL and lecture-based learning (LBL) in undergraduate pharmacology education.

**Methods:**

We systematically searched literature databases for comparative studies related to PBL pedagogy in the undergraduate pharmacology curriculum from the inception of the databases to December 2023. The obtained literature was screened according to the selection criteria, and Review Manager 5.4 was used for the meta-analysis of the included studies.

**Results:**

A total of 33 comparative studies involving 4425 undergraduate students were enrolled. The standardized mean differences (95% confidence intervals) of the examination scores and students’ self-rated scores on learning interest, comprehension of knowledge and thinking ability between PBL and LBL were calculated to be 2.03 (1.53–2.53), 0.50 (0.26–0.74), 0.69 (0.46–0.92), and 1.65 (1.21–2.09), respectively. The risk ratios of the proportion of satisfaction on improving students’ learning interest, comprehension of knowledge, thinking ability, self-study ability, and communication skills were calculated to be 2.08 (1.17–3.71), 1.84 (1.26–2.67), 1.42 (1.19–1.69), 1.44 (1.16–1.79), and 1.66 (1.22–2.27), respectively.

**Conclusions:**

The current evidence indicates that PBL is more effective than LBL in improving examination scores and student satisfaction in undergraduate pharmacology education.

## 1. Introduction

Pharmacology is an integrative discipline that deals with the interactions between drugs/natural products and living systems.^[1]^ It is one of the cornerstones of health science and research-based biomedical courses in higher education.^[2]^ The pharmacology curriculum plays a key role as a bridge between basic medicine, clinical medicine, and pharmacy and involves pharmacokinetics, pharmacodynamics, pharmacotherapeutics, and the application of drugs. The undergraduate stage is important for developing students’ knowledge and cultivating learning interest in pharmacology, and its teaching is critical and challenging. Undergraduate education in pharmacology has long been dominated by lecture-based learning (LBL). LBL is a traditional teaching pedagogy with teachers as the center, classroom teaching as the main approach, and passive knowledge imparting as the goal.^[3,4]^ However, the traditional LBL approach does not perform well in improving students’ academic performance or comprehensive ability.

Problem-based learning (PBL) is a student-centered teaching method that transfers the role of the teacher to that of the student and is based on self-motivated learning. It was first implemented in 1969 in a medical program at McMaster University in Canada,^[5]^ leaving aside the traditional LBL method and introducing student-centered learning. PBL enables students to construct their knowledge by solving problems. In this mode of learning, students can formulate assumptions and identify learning needs, thereby helping them better understand problems and achieve established learning goals.^[6]^ Previous studies have shown that students in PBL groups perform better on academic examinations than those in traditional LBL groups.^[7,8]^ In addition to improving academic performance, PBL makes the learning process enjoyable for both students and teachers,^[9]^ which may in turn lead to higher levels of motivation for all participants.

Nearly 50 years after the introduction of McMaster University concept, PBL pedagogy has been widely employed in pharmacology education. However, the benefits of PBL for undergraduate students have not been clearly demonstrated. Therefore, this study aimed to evaluate the effects of PBL in undergraduate pharmacology education through a systematic literature review and meta-analysis.

## 2. Methods

This meta-analysis was conducted in accordance with the Preferred Reporting Items for Systematic Reviews and Meta-Analyses guidelines to evaluate the effectiveness of PBL in improving the pharmacological learning of undergraduate students.^[10]^ Ethical approval was not required because this study did not disclose patient information and was only associated with related literature data.

### 2.1. Search strategy

Two reviewers (YX and YY) independently retrieved the literature from 6 databases, namely PubMed, Web of Science, Embase, Chinese Wanfang Database, China National Knowledge Infrastructure (CNKI), and Chinese VIP database, from database inception to December 2023. The details of the search strategy used for PubMed are as follows: ((problem-based learning[mesh]) or (problem-based learning[tiab]) or (problem based learning[tiab]) or (problem solving[mesh]) or (problem solving[tiab]) or (problem-based curriculum[tiab]) or (problem based curricula[tiab])) and ((pharmacology[tiab]) or (pharmacology [mesh])). Variations in this strategy were used to search the other databases.

### 2.2. Selection criteria

Studies that met the following inclusion criteria were included: (1) randomized controlled trials (RCTs) or controlled studies; (2) comparisons of PBL with the traditional LBL method; (3) participants: undergraduate students receiving pharmacology education; (4) outcome measurements of the PBL group or control group should be available; and (5) English or Chinese publications are both acceptable. The exclusion criteria were as follows: (1) repeated publications; (2) uncontrolled studies; (3) full text or outcome data not available; (4) disciplines unrelated to pharmacology; and (5) reviews, abstracts, theoretical discussions, case reports or meta-analyses.

### 2.3. Data extraction and outcome measures

Two reviewers independently extracted data based on the selection criteria listed above. Any differences in their data extraction results were resolved through discussions with a third investigator. The following information was extracted from all included studies: (1) first author and publication year, (2) country, (3) characteristics of participants, (4) sample size, and (5) outcome measurements. The primary outcome measure was the examination score. Other subjective outcome measurements based on responses to the questionnaires included students’ learning interest, comprehension of knowledge, learning efficiency, self-study ability, communication skills, and satisfaction.

### 2.4. Quality assessment

The Cochrane Collaboration’s tool was used to assess the risk of bias in the included studies: random sequence generation, allocation concealment, blinding of participants and outcome assessment, incomplete outcome data, selective reporting, and other biases.^[11]^ All items were checked and classified as having low, high, or unclear risk of bias.

### 2.5. Statistical analysis

This meta-analysis was conducted using Review Manager 5.4 software (The Cochrane Collaboration, London, UK). The outcomes were summarized as standardized mean differences (SMD) for continuous data and risk ratios (RR) for dichotomous data, with 95% confidence intervals (CI), calculated using the random or fixed-effects model. The heterogeneity assumption was weighed by using the *I*^2^ test. A random-effects model was adopted if *I*^2^ ≥ 50%. A fixed-effects model was adopted if *I*^2^ was <50%. SMD >0 indicates a higher score for the PBL method, and a 95% CI that did not contain 0 was deemed to be statistically significant. An RR > 1 indicates a higher incidence of students who agreed with the effectiveness of the PBL method, and a 95% CI that did not contain 1 was considered statistically significant.

## 3. Results

### 3.1. Study selection and basic characteristics

**Figure 1.**
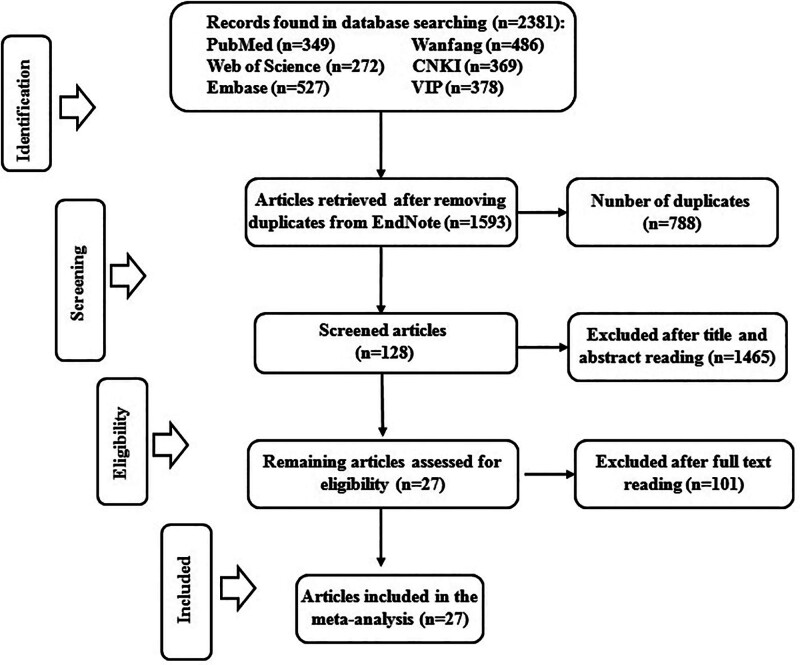
Flow chart of the literature screening and selection process.

Two thousand three hundred eighty-one articles were obtained from the primary literature search, of which 1593 remained after the removal of duplicates. After reviewing titles and abstracts, 128 articles were retained for further examination. According to the predefined selection criteria, 27 articles with 33 comparative studies that fulfilled the eligibility criteria were ultimately included in the meta-analysis after the full texts were read. The detailed literature screening process and results are shown in Figure 1.

### 3.2. Characteristics of the included studies

This meta-analysis included 27 articles containing a total of 33 comparative studies enrolling 4425 undergraduate students.^[12–38]^ The characteristics of the included studies are presented in Table 1. The majority of the studies were conducted in China (n = 21), followed by Germany (n = 7), Yemen (n = 2), the USA (n = 1), Portugal (n = 1), and the Netherlands (n = 1). The participants were mainly medical (21/33, 63.6%), pharmacy (7/33, 21.2%), and nursing students (3/33, 9.1%). The sample sizes ranged from 15 to 220.

**Table 1:** Characteristics of the included studies.

Author (year)	Country	Participant characteristics	Total number	PBL (n)	LBL (n)	Outcome measurements
Antepohl et al (1999)^[12]^	Germany	Fifth-semester medical students	112	55	57	①
Brinkman et al (2021)^[13]^	Portugal	Final-year medical students	90	54	36	①
De et al (1993)^[14]^	Netherlands	Fifth and sixth year medical students	203	103	100	①
Hassan et al (2000a)^[15]^	Yemen	Fourth-year medical students	60	33	27	①
Hassan et al (2000b)^[15]^	Yemen	Final-year medical assistant students	40	23	17	①
Herzig et al (2003a)^[16]^	Germany	Third-year medical students	112	55	57	①
Herzig et al (2003b)^[16]^	Germany	Third-year medical students	90	45	45	①
Herzig et al (2003c)^[16]^	Germany	Third-year medical students	32	17	15	①
Liang et al (2017)^[17]^	China	Third-year medical students and nursing students	50	25	25	①⑧⑨
Liang (2) et al (2017)^[18]^	China	Third-year medical students	80	40	40	①⑦⑧
Likic et al (2009)^[19]^	USA	Final-semester medical students	315	220	95	①
Michel et al (2002a)^[20]^	Germany	Third-year medical students	189	60	129	①
Michel et al (2002b)^[20]^	Germany	Third-year medical students	84	28	56	⑦⑧
Michel et al (2002c)^[20]^	Germany	Third-year medical students	142	40	102	⑦⑧
Song et al (2015)^[21]^	China	Pharmacy students in grade 2012	183	90	93	①②③④⑤⑥
Song et al (2008)^[22]^	China	Medical students in grade 2004	116	85	31	①③④⑤⑥
Yang et al (2018)^[23]^	China	Medical students in grade 2013	60	30	30	①⑨
Fu et al (2016)^[24]^	China	Medical students in grade 2013	188	54	134	①②
Zhou et al (2023)^[25]^	China	Medical students in grade 2020	188	95	93	①
Zhou et al (2012)^[26]^	China	Pharmacy students in grade 2007	68	34	34	①
Li et al (2019)^[27]^	China	Medical students in grade 2015	194	98	96	①④⑤⑥
Li et al (2011)^[28]^	China	Pharmacy students in grade 2006	108	54	54	①
Yang et al (2008)^[29]^	China	Nursing students in grade 2004	56	26	30	①
Liang et al (2008)^[30]^	China	Nursing students in grade 2006	179	89	90	①②④⑤
Wang et al (2008)^[31]^	China	Medical students in grade 2003	309	189	120	①
Zhao et al (2014a)^[32]^	China	Pharmacy students in grade 2007	127	80	47	①
Zhao et al (2014b)^[32]^	China	Pharmacy students in grade 2009	154	71	83	①④⑤⑥
Zhao et al (2015)^[33]^	China	Pharmacy students in grade 2010	140	70	70	①
Zhao et al (2021)^[34]^	China	Nursing students	300	150	150	①
Chen et al (2015)^[35]^	China	Medical students in grade 2012	145	82	63	①
Huo et al (2016)^[36]^	China	Medical students in grade 2013	113	56	57	①③④⑤⑥
Gao et al (2015)^[37]^	China	Pharmacy students in grade 2011 and 2012	118	60	58	①②③④⑥
Qin et al (2012)^[38]^	China	Medical students	80	40	40	②③④

① The examination score. ② The proportion of satisfaction on improving learning interest. ③ The proportion of satisfaction on improving comprehension of knowledge. ④ The proportion of satisfaction on improving thinking ability. ⑤ The proportion of satisfaction on improving self-study ability. ⑥ The proportion of satisfaction on improving communication skills. ⑦ Students’ self-rated scores on their learning interest. ⑧ Students’ self-rated scores on their comprehension of knowledge. ⑨ Students’ self-rated scores on their thinking ability.

LBL = lecture based learning, PBL = problem-based learning.

Regarding outcome measurements, 30 studies reported the students’ examination scores. The incidence of students who accepted the effects of the PBL method on improving their learning interest, comprehension of knowledge, thinking ability, self-study ability, and communication skills was reported in 5, 5, 8, 6, and 6 studies, respectively. Self-rated scores for improving their learning interest, comprehension of knowledge, and thinking ability were reported in 3, 4, and 2 studies, respectively.

### 3.3. Evaluation of methodological quality included in the study

**Figure 2.**
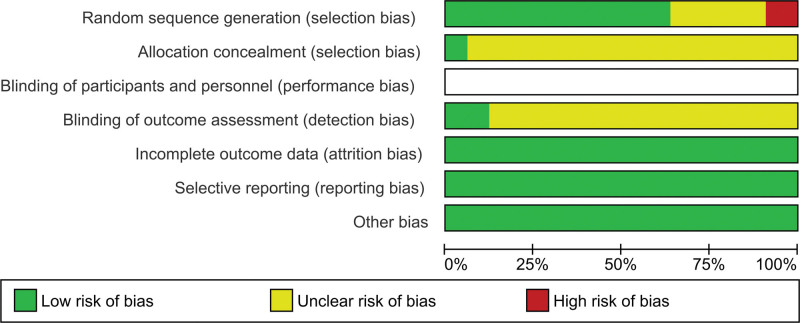
Risk of bias graph of included studies.

The Cochrane risk-of-bias tool was employed for quality assessment of the 33 included studies. For selection bias, a total of 21 (63.6%) studies used an appropriate random sequence generation method and were assessed as low risk, 3 (9.1%) comparative nonrandomized studies were rated as high risk, and 2 studies implemented an allocation concealment procedure and were assessed as low risk. Four studies reported the blinding method for outcome assessment and were rated as low risk. Given the characteristics of the teaching process, teachers and participants could not be blinded. Therefore, the performance bias was not applicable. All studies had complete data and were not selectively reported, and hence the attrition bias and reporting bias were rated as low risk. A summary of the assessed outcomes is shown in Figure 2.

### 3.4. Effect on improving students’ examination scores

**Figure 3.**
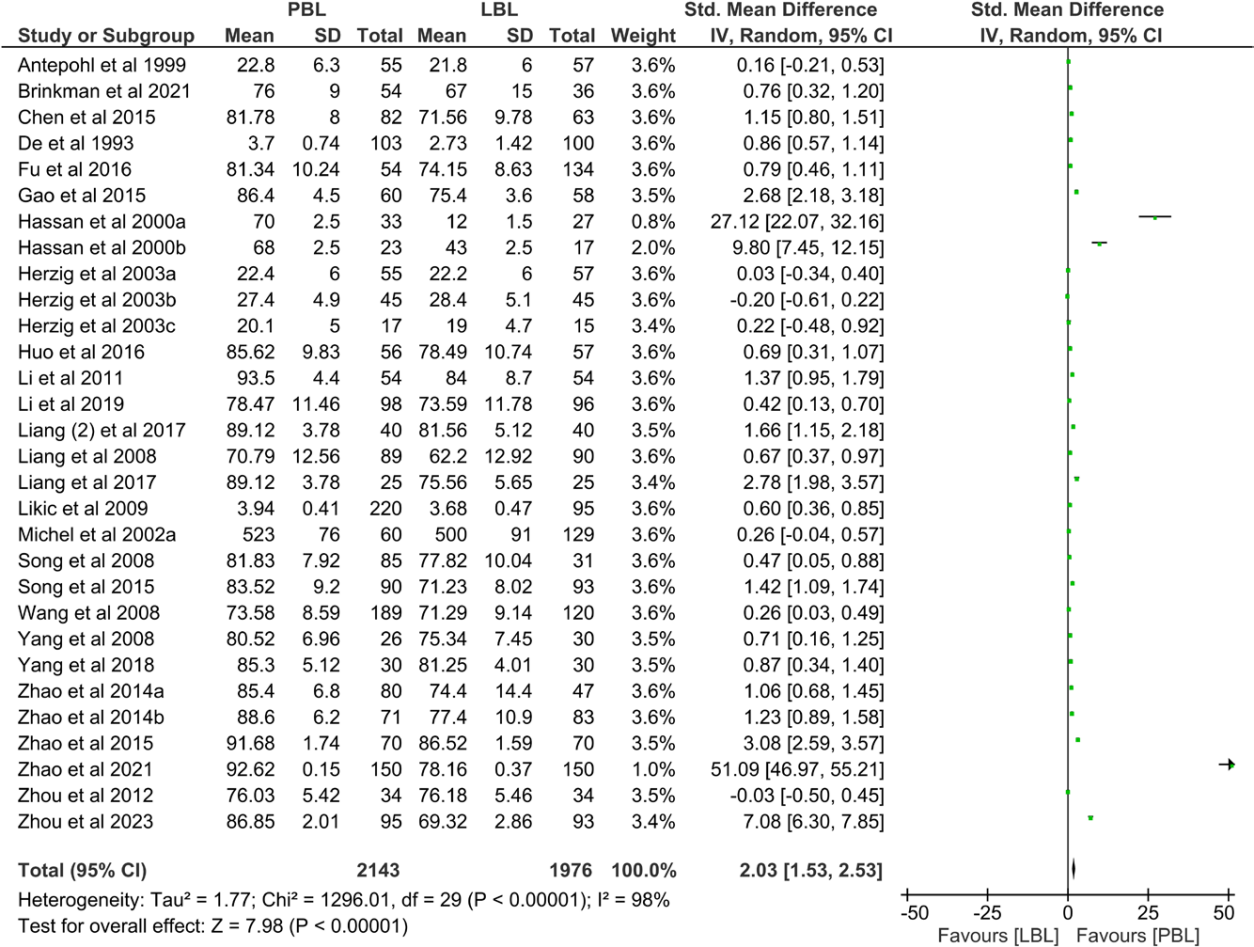
Forest plot of examination scores for PBL compared with LBL. CI = confidence interval, LBL = lecture-based learning, PBL = problem-based learning.

The examination score directly reflects the effectiveness of the PBL method in undergraduate pharmacology education, and serves as the primary outcome measure. Among the 33 included studies, 30 studies involving 4119 participants reported examination scores.^[12–37]^ The SMDs are shown in Figure 3. A random-effects model was adopted due to the existence of heterogeneity (*I*^2^ = 98%). Students receiving the PBL method in undergraduate pharmacology education tended to achieve higher examination scores in objective tests than those receiving the traditional LBL method (SMD = 2.03, 95% CI [1.53, 2.53], *P* < .00001). This demonstrates that PBL pedagogy is better than LBL in improving the examination scores of the pharmacology curriculum for undergraduate education.

Owing to the substantial heterogeneity observed between studies, a sensitivity analysis was carried out to reassess the reliability of the results by sequentially excluding individual studies. However, the source cannot be definitively attributed to a single study. This pooled analysis with heterogeneity was similar to that of a previously published meta-analysis.^[39]^ We also performed subgroup analyses with 3 variables-student major, publication year, and student number-to identify the sources of heterogeneity. The results are presented in Table 2. High *I^2^* values were still observed in the subgroup analyses, suggesting that heterogeneity remained. Through subgroup analysis, we also found that the PBL teaching method was beneficial for students from different majors, including medicine (SMD = 1.33, 95% CI [0.85, 1.81], *P* < .00001), pharmacy (SMD = 1.54, 95% CI [0.88, 2.19], *P* < .00001), and nursing (SMD = 15.91, 95% CI [9.77, 22.05], *P* < .00001).

**Table 2:** Subgroup analysis of examination scores for PBL compared with LBL.

Variable	No. of studies	No. of students	SMD	95% CI	Heterogeneity (*I*^2^)	Model
Student major						
Medicine	19	2636	1.33	0.85–1.81	<0.00001 (96%)	Random
Pharmacy	7	898	1.54	0.88–2.19	<0.00001 (94%)	Random
Nursing	3	535	15.91	9.77–22.05	<0.00001 (100%)	Random
Publication year						
Before 2010	13	1813	0.81	0.36–1.26	<0.00001 (94%)	Random
After 2010	17	2306	2.87	2.05–3.69	<0.00001 (98%)	Random
Student number						
≤100	10	626	2.25	1.25–3.25	<0.00001 (96%)	Random
101–200	16	2366	1.37	0.86–1.88	<0.00001 (97%)	Random
˃200	4	1127	8.10	5.80–10.41	<0.00001 (99%)	Random

CI = confidence interval, SMD = standardized mean difference.

### 3.5. Effects on improving students’ learning interest

Five studies involving 748 students reported satisfaction with improving students’ learning interest.^[21,24,30,37,38]^ Compared with LBL, a larger percentage of participants thought that the PBL method had a greater influence on improving learning interest (76.0% in the PBL group vs 41.4% in the LBL group, RR = 2.08, 95% CI [1.17, 3.71], *P* = .01, *I*^2^ = 97%). In addition, 3 studies reported students’ self-rated scores on their learning interest in PBL and LBL groups.^[18,20]^ The learning interest scores were significantly higher in the PBL group than in the LBL group (SMD = 0.50, 95% CI [0.26, 0.74], *P* < .0001, *I*^2^ = 0%). The above results demonstrate that PBL pedagogy is better than LBL in improving students’ learning interest in pharmacology (Table 3).

**Table 3:** Statistical data of the current meta-analysis.

Outcome measurements	No. of studies	No. of participants			Incidence			
		PBL	LBL	*P*	SMD/RR	95% CI	Heterogeneity (*I*^2^)	Model
The examination score	30	2143	1976	<.00001	2.03	1.53–2.53	<0.00001 (98%)	Random
The proportion of satisfaction on improving:								
Learning interest	5	333	415	.01	2.08	1.17–3.71	<0.00001 (97%)	Random
Comprehension of knowledge	5	331	279	.001	1.84	1.26–2.67	<0.0001 (85%)	Random
Thinking ability	8	589	548	<.0001	1.42	1.19–1.69	<0.00001 (83%)	Random
Self-study ability	6	489	450	.0008	1.44	1.16–1.79	<0.0001 (84%)	Random
Communication skills	6	460	418	.001	1.66	1.22–2.27	<0.00001 (85%)	Random
Students’ self-rated scores on:								
Learning interest	3	108	198	<.0001	0.50	0.26–0.74	0.98 (0%)	Fixed
Comprehension of knowledge	4	133	223	<.00001	0.69	0.46–0.92	0.65 (0%)	Fixed
Thinking ability	2	55	55	<.00001	1.65	1.21–2.09	0.26 (22%)	Fixed

CI = confidence interval, LBL = lecture-based learning, PBL = problem-based learning, RR = risk ratio, SMD = standardized mean difference.

### 3.6. Effects on improving students’ comprehension of knowledge

A total of 5 studies involving 610 students revealed the incidence of students who accepted the effects of PBL on improving their comprehension of knowledge.^[21,22,36–38]^ Compared with LBL, a higher proportion of students believed that the PBL method had a greater impact on improving their comprehension of knowledge (71.0% in the PBL group vs 41.2% in the LBL group, RR = 1.84, 95% CI [1.26, 2.67], *P* = .001, *I*^2^ = 85%). Moreover, 4 studies reported students’ self-rated scores on their comprehension of knowledge in PBL and LBL groups.^[17,18,20]^ The SMD with 95% CIs for self-reported knowledge comprehension scores was calculated to be 0.69 (0.46, 0.92). These findings prove that the PBL method is better than LBL in the improvement of students’ knowledge comprehension in the undergraduate pharmacology curriculum (Table 3).

### 3.7. Effects on improving students’ thinking ability

Eight studies involving 1137 students analyzed the proportion of satisfaction in improving students’ thinking ability.^[21,22,27,30,32,36–38]^ Compared with LBL, a higher proportion of participants felt that the PBL method had a greater influence on improving their thinking ability (74.0% in the PBL group vs 56.9% in the LBL group, RR = 1.42, 95% CI [1.19, 1.69], *P* < .0001, *I*^2^ = 83%). Two studies reported students’ self-rated scores on their thinking ability in PBL and LBL groups.^[17,23]^ Similarly, students’ self-rated scores on their thinking ability were significantly higher in the PBL group than in the LBL group (SMD = 1.65, 95% CI [1.21, 2.09], *P* < .00001, *I*^2^ = 22%). These findings demonstrate that the PBL method is better than LBL in improving students’ thinking ability in the undergraduate pharmacology curriculum (Table 3).

### 3.8. Effects on improving students’ self-study ability

A total of 6 studies involving 939 students reported the incidence of students who accepted the effects of PBL on improving their self-study ability.^[21,22,27,30,32,36]^ We found that a higher proportion of participants thought that the PBL method strongly influenced their self-study ability (72.8% in the PBL group vs 54.7% in the LBL group, RR = 1.44, 95% CI [1.16, 1.79], *P* = .0008, *I*^2^ = 84%) when compared to the LBL method in random-effects model. This demonstrates that the PBL method is better than LBL in the improvement of students’ self-study ability in the pharmacology curriculum for undergraduate education (Table 3).

### 3.9. Effects on improving students’ communication skills

Regarding the incidence of students who were satisfied with improving their thinking ability, 6 studies involving 878 students were analyzed, of whom 460 received the PBL method.^[21,22,27,32,36,37]^ Compared with LBL, a higher proportion of students believed that PBL had a greater impact on improving communication skills thinking (64.8% in the PBL group vs 44.3% in the LBL group, RR = 1.66, 95% CI [1.22, 2.27], *P* = .001, *I*^2^ = 85%).This indicates that the PBL method is better than LBL in the improvement of students’ communication skills in the undergraduate pharmacology curriculum (Table 3).

### 3.10. Publication bias

**Figure 4.**
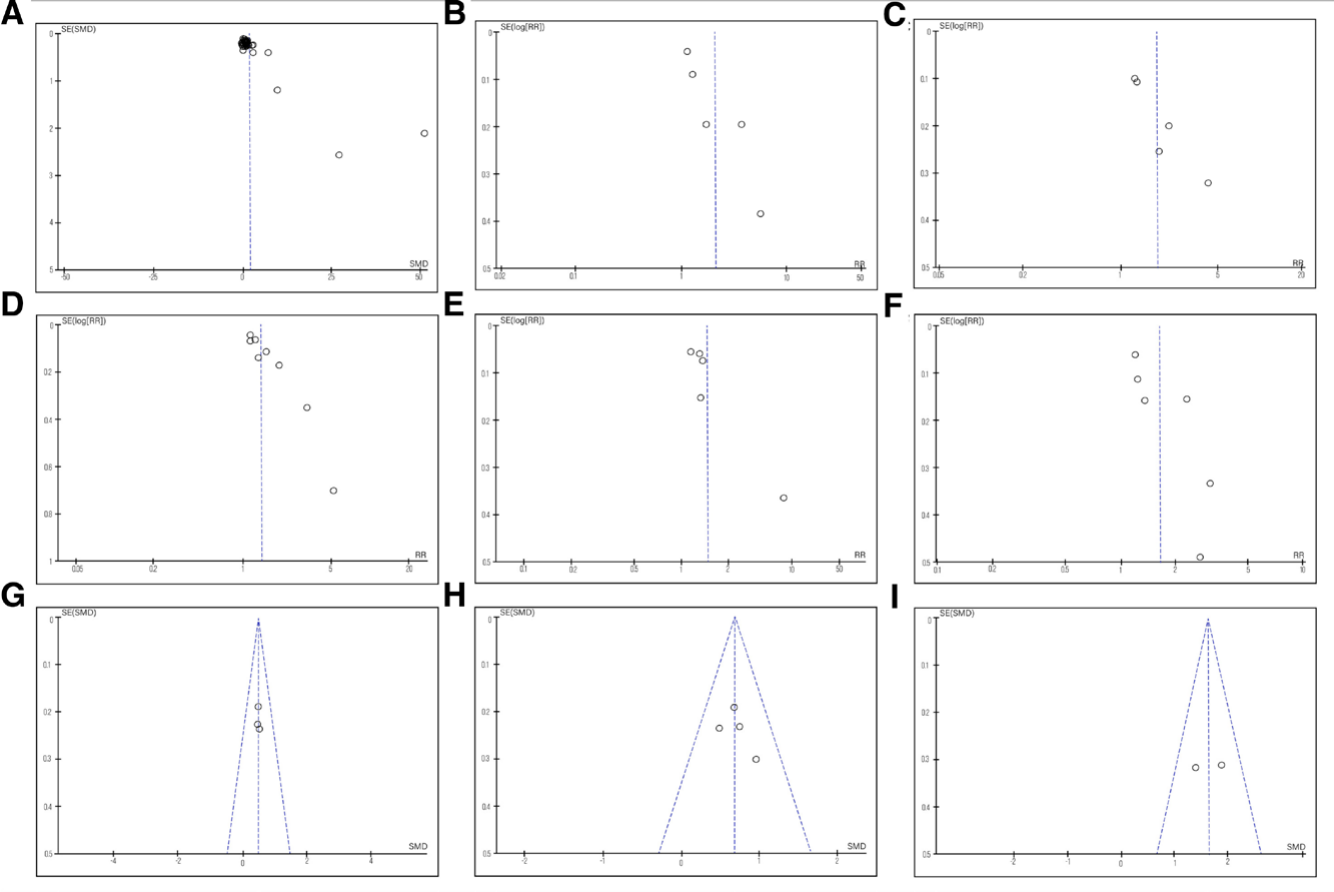
Publication bias of (A) examination scores, (B) proportion of satisfaction on improving learning interest, (C) proportion of satisfaction on improving comprehension of knowledge, (D) proportion of satisfaction on improving thinking ability, (E) proportion of satisfaction on improving self-study ability, (F) proportion of satisfaction on improving communication skills, (G) students’ self-rated scores on learning interest, (H) students’ self-rated scores on comprehension of knowledge, and (I) students’ self-rated scores on thinking ability. RR = risk ratio, SE = standard error, SMD = standardized mean difference.

Funnel plots of examination scores, proportion of satisfaction on improving learning interest, comprehension of knowledge, thinking ability, self-study ability, and communication skills, and students’ self-rated scores on learning interest, comprehension of knowledge, and thinking ability were used to evaluate publication bias. Not all funnel plots are totally symmetrical, indicating potential publication bias exists (Fig. 4A–I).

## 4. Discussion

In recent decades, increasing research has highlighted the effectiveness of PBL pedagogy in pharmacology education. However, to our knowledge, no systematic analysis has proven that PBL pedagogy is more effective than traditional LBL pedagogy in undergraduate pharmacology teaching. Based on this, a large-scale and comprehensive meta-analysis of PBL teaching method was carried out. We systematically searched literature databases and included 27 articles containing 33 comparative studies involving 4425 undergraduate students. The results demonstrated that PBL was significantly superior to LBL in terms of improving examination scores, students’ learning interest, comprehension of knowledge, thinking ability, self-study ability, and communication skills.

PBL is a student-centered and self- directed learning model that follows the process of identifying problems, gathering necessary information, and developing solutions.^[40]^ In contrast to LBL, PBL requires students to have the ability to solve practical problems by self-learning through text, rather than merely passively accepting knowledge from teachers.^[3,4]^ In the PBL teaching model, students learn and discuss in small groups, facilitating teamwork and promoting their communication skills. PBL can effectively stimulate students’ learning interests and fully mobilize and utilize their enthusiasm for learning. In addition, PBL has been used for the curricular integration of different subjects in several medical schools.^[41]^ This integration can place the basic science material into a clinical setting thereby increasing their utility.^[42,43]^ Pharmacology is a curriculum that requires the integration of basic and clinical science. The current meta-analysis found that the PBL method was more effective than the LBL method in improving examination scores and student satisfaction in undergraduate pharmacology education. This finding is consistent with the results reported in previous meta-analyses of PBL methods in other curricula.^[8,44]^

Although the PBL method has obvious advantages in undergraduate pharmacology teaching, it is difficult to implement. The data from some of these included studies do not provide evidence that PBL has a beneficial effect on factual knowledge.^[16,26]^ Even when students were exposed to advanced learning in pharmacology, the control group showed an opposite tendency to perform better.^[16]^ Perhaps, these students were more comfortable with traditional teaching methods, which they had already perceived in their previous learning. One disadvantage of the PBL method is that students have to spend more time and energy in the process of analyzing and solving problems and are more likely to miss some key knowledge. The PBL method requires lecturers to address appropriate scientific problems to help students understand pharmacology. Therefore, it is highly desirable for teachers to be comprehensive. Additional human and material resources are needed for the implementation of PBL. Teachers lacking experience in leading self-studies require more training. Some lecturers may be uncomfortable with this method and therefore cannot apply it properly.

Several limitations need to be taken into consideration. Potential publication bias and language bias may have been introduced into the meta-analysis because it was limited to including published English and Chinese studies. The implementation of blinding in the participants was not performed because of the open environment of the teaching process. Two types of outcome measurements—examination scores and questionnaire responses—were collected and analyzed. In fact, different studies have reported different types of questionnaires; however, we only combined data from studies that included the same items. The investigation of the effects of the PBL method focuses on students’ learning interest, comprehension of knowledge, thinking ability, self-study ability, and communication skills. High-quality evidence and a variety of standardized evaluation methods are necessary for an in-depth and comprehensive evaluation of the effectiveness of PBL pedagogy.

## 5. Conclusions

In conclusion, the current evidence indicates that the PBL method in undergraduate pharmacology teaching is more effective than LBL in improving examination scores and students’ learning interest, comprehension of knowledge, thinking ability, self-study ability, and communication skills, thus providing evidence for the implementation of PBL in undergraduate pharmacology education. It is imperative that pharmacology pedagogues further introduce and promote PBL pedagogy among undergraduate students.

## Author contributions

**Conceptualization:** Yongyu Yang.

**Data curation:** Xiding Yang, Yongyu Yang.

**Formal analysis:** Xiding Yang, Yongyu Yang.

**Investigation:** Xiding Yang, Miao Yan.

**Methodology:** Xiding Yang, Zehua Yang, Sijia Ma, Yongyu Yang.

**Software:** Xiding Yang, Zehua Yang, Sijia Ma.

**Writing – original draft:** Xiding Yang, Yongyu Yang.

**Writing – review & editing:** Xiding Yang, Zehua Yang, Sijia Ma, Miao Yan, Yongyu Yang.
